# The advective origin of an under-ice spring bloom in the Arctic Ocean using multiple observational platforms

**DOI:** 10.1007/s00300-018-2278-5

**Published:** 2018-02-13

**Authors:** Geir Johnsen, Marit Norli, Mark Moline, Ian Robbins, Cecilie von Quillfeldt, Kai Sørensen, Finlo Cottier, Jørgen Berge

**Affiliations:** 1 Centre for Autonomous Marine Operations and Systems, Department of Biology, Norwegian University of Technology and Science (NTNU), 7491 Trondheim, Norway; 2 Department of Arctic Biology, University Centre on Svalbard (UNIS), 9171 Longyearbyen, Norway; 3 Department of Arctic and Marine Biology, UiT The Arctic University of Norway, 9037 Tromsø, Norway; 4 Section of Marine Biogeochemistry and Oceanography, Norwegian Institute of Water Research (NIVA), 0349 Oslo, Norway; 5 School of Marine Science and Policy, University of Delaware, Newark, DE 19958 USA; 6 California Polytechnic State University, San Luis Obispo, CA 93407 USA; 7 Department of Environment and Mapping, Norwegian Polar Institute (NP), 9296 Tromsø, Norway; 8 Scottish Association for Marine Science (SAMS), Oban, Argyll, PA37 1QA UK

**Keywords:** Sea-ice algae, Under-ice bloom, Phytoplankton, Photoacclimation, Advection of cells, Arctic, Satellite, Autonomous underwater vehicle, Chlorophyll *a*, Pulse amplitude modulated fluorescence (PAM)

## Abstract

Under-ice blooms of phytoplankton in the Chukchi Sea have been observed, with strong implications for our understanding of the production regimes in the Arctic Ocean. Using a combination of satellite remote sensing of phytoplankton biomass, in situ observations under sea ice from an autonomous underwater vehicle (AUV), and in vivo photophysiology, we examined the composition, magnitude and origin of a bloom detected beneath the sea ice Northwest of Svalbard (Southern Yermak Plateau) in May 2010. In situ concentration of up to 20 mg chlorophyll a [Chl *a*] m^−3^, were dominated by the northern planktonic spring species of diatoms, *Thalassiosira nordenskioeldii*, *T. antarctica* var. *borealis*, *Chaetoceros socialis* *species complex* and *Fragilariopsis oceanica*. These species were also found south of the marginal ice zone (MIZ). Cells in the water column under the sea ice were typically high-light acclimated, with a mean light saturation index (*E*_*k*_) of 138 μmol photons m^−2^ s^−1^ and a ratio between photoprotective carotenoids (PPC) and Chl *a* (w:w) of 0.2. Remotely sensed data of [Chl *a*] showed a 32,000 km^2^ bloom developing south of the MIZ. In effect, our data suggest that the observed under-ice bloom was in fact a bloom developed in open waters south of the ice edge, and that a combination of northward-flowing water masses and southward drifting sea ice effectively positioned the bloom under the sea ice. This have implications for our general understanding of under-ice blooms, suggesting that their origin and connection with open water may be different in different regions of the Arctic.

## Introduction

Sea ice plays a dual role in the control of high-latitude phytoplankton blooms by influencing both light shading and stratification (Arrigo et al. 2012; Assmy et al. 2017; Kauko et al. 2017). Importantly, primary production (PP) in regions with an annual ice cover comes from two main sources, i.e. ice-associated and open water blooms (Søreide et al. 2010). These two main sources of PP have traditionally been considered as temporally and spatially distinct. It has been suggested that withdrawal of ice will generally lead to an increase in PP (Drinkwater 2011; Slagstad et al. 2015), and that the nutrient-dependent new production may not increase proportionally with increasing light intensity due to insufficient access to essential nutrients. Recently, new reports of a “third” source of PP were presented, under-ice phytoplankton blooms (see e.g. Arrigo et al. 2012). A phytoplankton bloom beneath the sea ice is a phenomenon that is poorly understood, they are hard to detect and effectively invisible from satellites, and they occur in habitats that are logistically hard to sample. Yet, there are several reports documenting their existence (Gradinger 1996; Mundy et al. 2009; Boetius et al. 2013; Assmy et al. 2017), but few that estimate their role in the total productivity and carbon budget of the Arctic. In the Chukchi Sea, Arrigo et al. (2012, 2014) estimated that 90% of the total PP occurred under sea ice. Importantly, as most current models rely on remote sensing for input and validation of phytoplankton biomass, occurring beneath an ice cover, phytoplankton biomass in an under-ice bloom is at best poorly represented in contemporary modelling efforts (Vancoppenolle et al. 2013). Under-ice blooms are therefore important to map and understand, as they may represent a significant component that is missing in the total production regime of the Arctic Ocean. Also, the sea-ice zone has been identified as the region with largest model uncertainty regarding Arctic PP and biophysical interactions (Assmy et al. 2017; Kauko et al. 2017). Knowing that under-ice blooms have been reported from across the central Arctic Ocean and surrounding shelf seas, yet not taken into account in contemporary modelling efforts, there is a clear and important gap in knowledge that needs to be filled: A quantitative and mechanistic understanding of the processes that control PP in ice-covered waters.

The concentration of Chlorophyll a, [Chl *a*], is the main source of information to detect, map and monitor phytoplankton biomass (review in Johnsen et al. 2011a, b). The differences in water mass-related effects on phytoplankton dynamics in the Svalbard area, often seen as the balance between the warm and saline water masses of Atlantic origin versus the colder and fresher water masses of Arctic origin, is reflected in the differences in biodiversity and physiological status of phytoplankton (Leu et al. 2011; Pettersen et al. 2011; Hovland et al. 2013; Hancke et al. 2014; Hovland et al. 2014). The key properties of the water masses and ice conditions in this region are well documented in terms of current speed and direction, temperature, salinity and density (Rudels et al. 2005; Ingvaldsen and Loeng 2009; Lind and Ingvaldsen 2012; Assmy et al. 2017).

Pelagic phytoplankton blooms in the MIZ are typically initiated from mid-April to June in the waters west of Spitsbergen and in the Barents Sea, and are associated with both Arctic cold water species and/or temperate species (McMinn and Hegseth 2007; Sakshaug et al. 2009a, b). In contrast, blooms in the perennial ice zone (PIZ) are generally thought of as mainly being associated with ice algae, with irradiance in the water column below ice too low to support a pelagic bloom (Sakshaug et al. 2009b; Berge et al. 2015). Recently, Arrigo et al. (2012) provided new and important insights into the occurrence and development of pelagic blooms under Arctic sea ice. A recent report from the same area as this study (NW of Svalbard), shows the dynamics between sea ice, the local current systems and a phytoplankton bloom dominated by *Phaeocystis pouchetii* (Assmy et al. 2017).

The light regime (light climate) is a function of three variables, the irradiance (*E*) in the PAR region, *E*_PAR_ in μmol quanta m^−2^ s^−1^ (photosynthetic active radiation, 400–700 nm), the spectral irradiance, *E* (*λ*) in μmol quanta m^−2^ s^−1^ nm^−1^, and day length in hours (Falkowski and Raven 1997; Sakshaug et al. 1997, 2009b). Photoacclimation (short-term physiological adjustment of growth rate to light climate) is divided in short- (seconds–minutes) and long-term (hours–days) responses (MacIntyre et al. 2000; Brunet et al. 2011; Valle et al. 2014). Short-term photoacclimation includes the xanthophyll cycle for photoprotection, an important part of non-photochemical quenching (NPQ). Long-term photoacclimation is defined as change in ratio between light-harvesting pigments (LHP) and photoprotective carotenoids (PPC), photosynthetic parameters, enzymatic activities involved in photosynthesis and respiration, and changes in cell volume and chemical composition (Brunet et al. 2011).

Qualitative and quantitative pigment analyses of chlorophylls and carotenoids by high performance liquid chromatography (HPLC) can be used for chemotaxonomical and photoacclimation information. Different pigment groups can be identified (Johnsen and Sakshaug 2007; Roy et al. 2011) such as the carotenoid fucoxanthin (spring blooms of diatoms), Chlorophyll *c*_3_ (prymnesiophytes such as *Emiliania huxleyi* and *Phaeocystis pouchetii*) and small prasinoxanthin-containing prasinophytes, typical for this area (Pettersen et al. 2011). Likewise, HPLC-isolated pigments comprise information of light-harvesting pigments (LHP) and photoprotective carotenoids (PPC), (Johnsen and Sakshaug 2007; Brunet et al. 2011; Johnsen et al. 2011a). The degradation state of chlorophylls can be also used to indicate the physiological state of the phytoplankton cells, i.e. pre-bloom, bloom and post-bloom state (Johnsen and Sakshaug 2000; Roy et al. 2011).

Through photoacclimation, the cells are optimizing the light harvesting properties, the corresponding photosynthetic electron transport rates (ETR, see Materials and Methods) and pigment functions (light harvesting versus photoprotective) to provide maximum growth rates (Falkowski and Raven 1997; Sakshaug et al. 1997; Brunet et al. 2011). Chlorophyll a fluorescence-based photosynthetic relative ETR rates (rETR), are typically measured by means of PAM (Genty et al. 1989; Kromkamp and Forster 2003; Hancke et al. 2008a, b) or by fast repetition rate fluorometer, FRRF (Kolber and Falkowski 1993; Suggett et al. 2009). The photoacclimation status of phytoplankton can be identified by looking at the photosynthetic parameters termed maximum light utilization coefficient (*α*_ETR_, similar to α for ^14^C-based *P* vs. *E* curves), Chl *a* fluorescence-based maximum rETR rate, rETR_max_ (similar to maximum photosynthetic rate, *P*_max_ for ^14^C-based *P* vs. *E* curves), and the corresponding light saturation parameter (*E*_*k*_, = rETR_max_/*α*_ETR_ in μmol quanta m^−2^ s^−1^) (Behrenfeld et al. 2004; Hancke et al. 2008b; Schuback et al. 2016).

Using *E*_*k*_ we can divide cells into high-light- (HL, cells growing in “surface open waters”) and low-light- (LL, cells grown “under sea ice” or deep waters) acclimated cells (Sakshaug et al. 2009b). The in situ *E*_*k*_ (μmol photons m^−2^ s^−1^) of spring bloom forming phytoplankton are typically 50–60 μmol photons m^−2^ s^−1^ at 0–10 m and 30–50 m depth, respectively in May–June (numbers from the Barents Sea using ^14^C-incubated cells, at latitudes comparable to the west of Spitsbergen at 78°N (reviewed by Sakshaug et al. 2009b).

To address our key scientific question “are the observed under-ice phytoplankton blooms a result of local production, or are most of the algal cells transported from open and well-lit waters south of the Marginal Ice Zone (MIZ)”, we combined information of phytoplankton biomass [Chl *a*] data from: Satellite (open water bloom south of ice edge covering 32,000 km^2^), an AUV deployed under sea ice [providing areal coverage of under-ice light transmission patchiness, (Chl *a*), temperature and salinity; horizontally 80,000 m^2^ and vertically 16,500 m^2^], vessel-based in situ profiles and corresponding work on cells in the laboratory (in vivo and in vitro). The major “pro” of using polar orbiting satellites equipped with ocean colour multispectral imagers for [Chl *a*] are the covering of large areas while the “cons” are that the information is restricted to water surface (upper 1–2 m), dependent on illumination from the sun and absence of clouds (review in Johnsen et al. 2011b). Autonomous underwater vehicles (propelled AUV’s) may cover the [Chl *a*] vertically and horizontally below 1 m depth and under sea ice, in contrast to satellite-derived data.

**Fig. 1:**
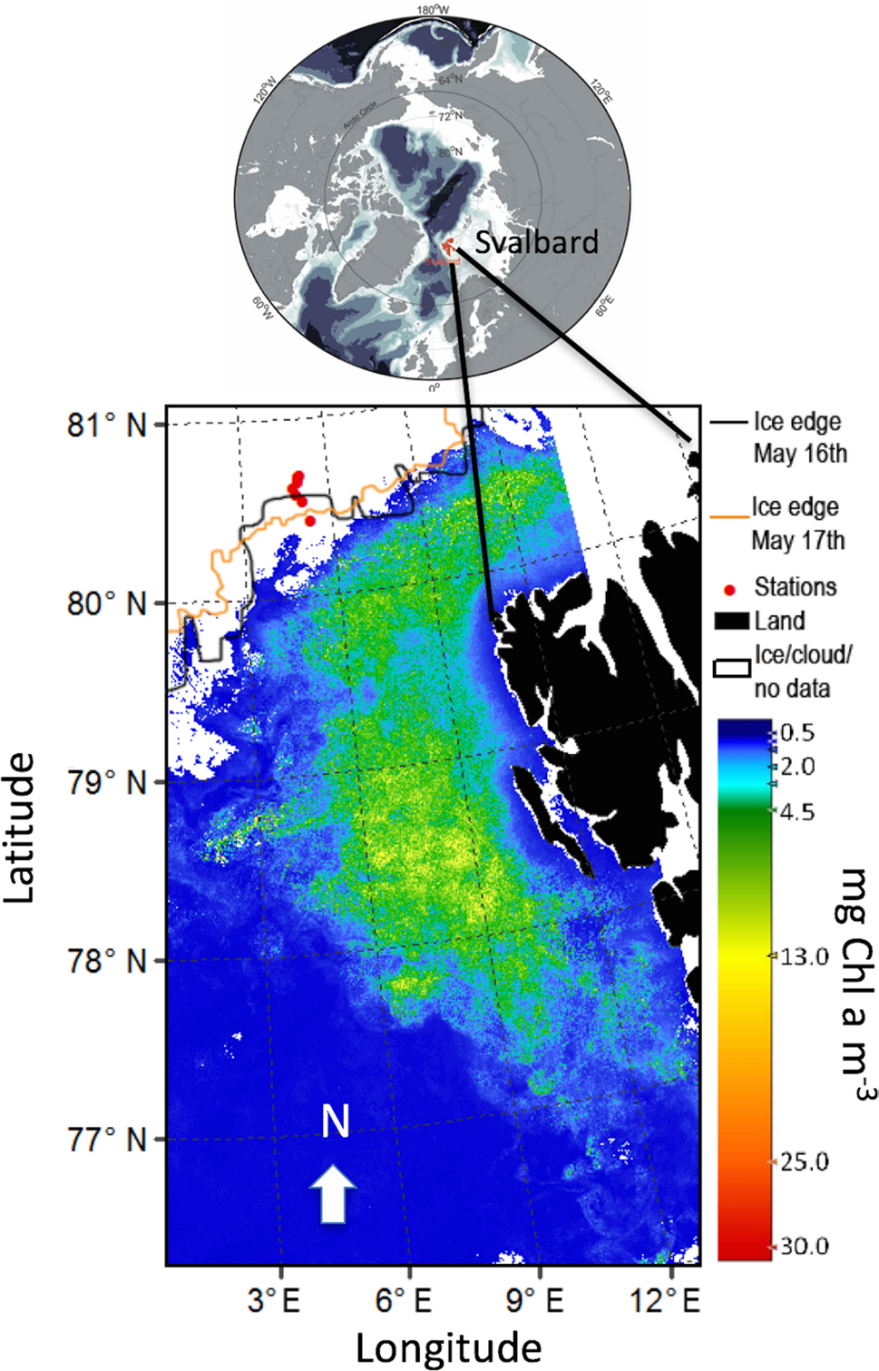
Remotely sensed sea surface phytoplankton bloom covering 32,000 km^2^ (green–yellow area), defined as [Chl *a*] > 4 mg m^−3^, in May 2010 (mean values) W and N of the Svalbard archipelago (black area). The phytoplankton biomass (colour bar in mg Chl *a* m^−3^), sea-ice distribution (white areas) with ice stations (red circles, northernmost stations at 31 km north of MIZ). The ice edge at 16 and 17 May indicated a southward ice movement during the campaign. Note that the southernmost station was in open waters during the actual transect. Image based on Envisat satellite with MERIS multispectral imager. For details, see Materials and methods

Based on this, we report on an under-ice bloom consisting of HL-acclimated cells in the Eurasian sector of the Arctic Ocean (southern part of the Yermak Plateau, Fig. 1) resulting from northward advection of water masses. This study shows that by combining information from three observational platforms (satellite, AUV and ship) we obtain a “picture” of overall biomass in both open waters (satellite) and under sea ice (AUV and ship). The ship-based CTD equipped with an in situ Chl *a* fluorometer and Niskin bottles for water sampling (phytoplankton) made it possible to study the taxa diversity and photoacclimation status (photosynthetic parameters) in addition to pigment speciation and function of the living cells sampled under the sea ice.

## Materials and methods

**Fig. 2:**
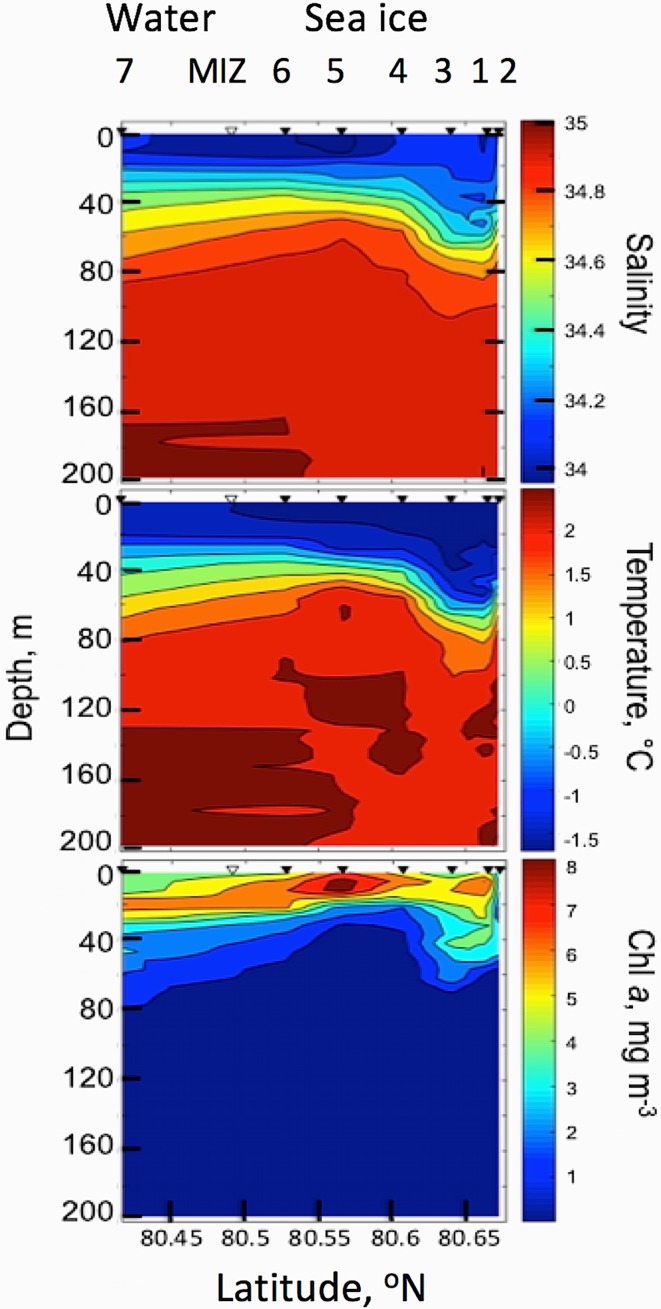
Ship-based under-ice 0–200 m vertical distribution of salinity (upper panel), temperature (mid panel) and [Chl *a*] (lower panel) through a transect line of 31 km north of the MIZ. Sea-ice stations 1–6, station 3 was the site for AUV survey, station 1–2 northernmost stations (right side), MIZ is the marginal ice zone and open water (station 7, southernmost)

The measurements were made to the north and south of the Marginal Ice Zone (MIZ) north of Svalbard 15–18 May 2010 (Fig. 1, Table 1). During sampling, the MIZ was at station (St) 6, St 7 in open waters (Fig. 1–2, Table 1) and the sea-ice coverage at St 1–5 was close to 100% with an ice thickness of 1.1–1.9 m and with some leads present. At station 1–3 the ice cores were ranging from 122 cm ± 12 (*n* = 11, SD) to 146 cm ± 40 (*n* = 8, SD). In addition, the corresponding snow depth on top of sea ice were ranging from 23 to 30 cm (*n* = 16). Ice cores were melted for ice algal analyses in cooling lab at 4°C in research vessel under dim light.

**Table 1:** Overview of ice station 1–7 with date, time of day (local time), latitude, longitude, CTD and Chl *a* fluorometer vertical transect from 0 to 200 m depth (all stations) from RV Helmer Hanssen, depth of water samples for pigment (all pigments by HPLC and corresponding in vitro Chl *a* analyses) and in vivo photosynthesis

Station	Date May	Time	Latitude	Longitude	Water sample depths (m)	Photosynthesis sample depths (m)	Pigments sample depths (m)	AUV sample depths (m)
1	15	10–13	80.665°N	5.048°E	2, 10	2, 10	2, 10	
2	15	22–01	80.673°N	5.100°E	2, 10, 40	2, 10, 40	2, 10, 40	
3	16	10–18	80.640°N	5.024°E	10, 45	10, 45	10, 45	5–55
4	16	23–24	80.607°N	4.841°E				
5	17–18	24–01	80.566°N	4.918°E				
6	17–18	24–01	80.528°N	5.097°E				
7	18	03–04	80.416°N	5.307°E				

Downwelling irradiance *E*_PAR_ and vertical net hauls of phytoplankton (both from surface to 50 m depths) were carried immediately after CTD profiles

### Observational platforms and sensors

Envisat satellite data from the MEdium resolution imaging spectrometer (MERIS) were used to identify and map the extent of the phytoplankton bloom (Chl *a*, mg m^−3^) south of the MIZ (Fig. 1–2) through a monthly mean [Chl *a*] map of the area. All available reduced resolution data (MERIS-RR) from May 2010 with a pixel size of 1 km from the MERIS archive at Brockmann Consults were used. The Algal-1 Chl *a* product algorithm from the 3rd reprocessing was processed using the software BEAM version 5.0.1 to produce the L3 binning product.

**Fig. 3:**
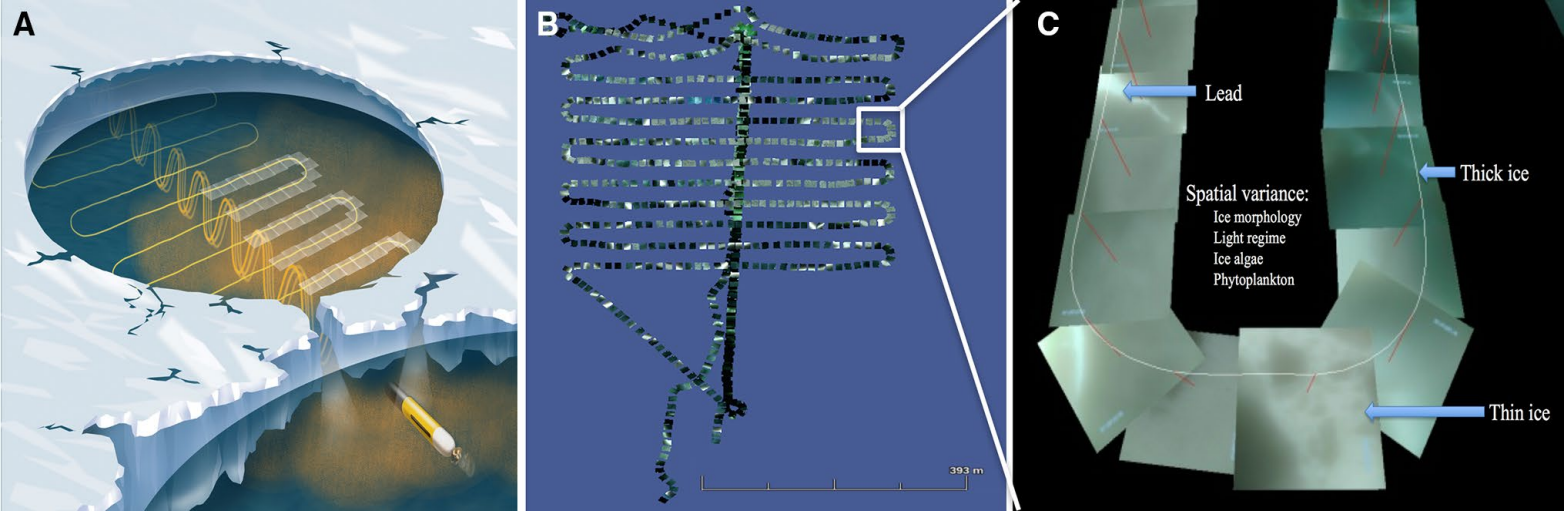
AUV-based under sea-ice photomosaic images at 10 m depth (horizontal survey, covering 80,000 m^2^) under the sea ice the 16th May 2010 at 11–12:15 local time at ice station 3. Deployment at 80°640′ N, 5°024′ E at average speed of 1.5 m s^−1^. At noon, under-ice E was between 1.2 and 2.1 μmol photons m^−2^ s^−1^ at 10 m depth (St 1–3, Fig. 4). At 22:00, E was typically about 30% of noon values (see results).** a** Schematic image of underwater AUV photomosaic images of underside of sea ice with camera facing upwards at 10 m depth during horizontal transect grid line. Indications on photomosaic images along track line shown on right side. White frame indicates area in** b** and** c**.** b** Complete photomosaics along grid line at 10 m depth.** c** Details (from** b**, white frame) of geo-tagged photomosaic images of under-ice light climate and ice morphology as a function of thick sea ice (dark picture frames), leads and thin ice (bright frames), blue areas indicating melt ponds (blue frames) and areas with high [Chl *a*] (green frames, indicating ice algae and phytoplankton). Corresponding horizontal and vertical AUV transect lines for Chl *a*, T and S in Fig. 5

**Fig. 4:**
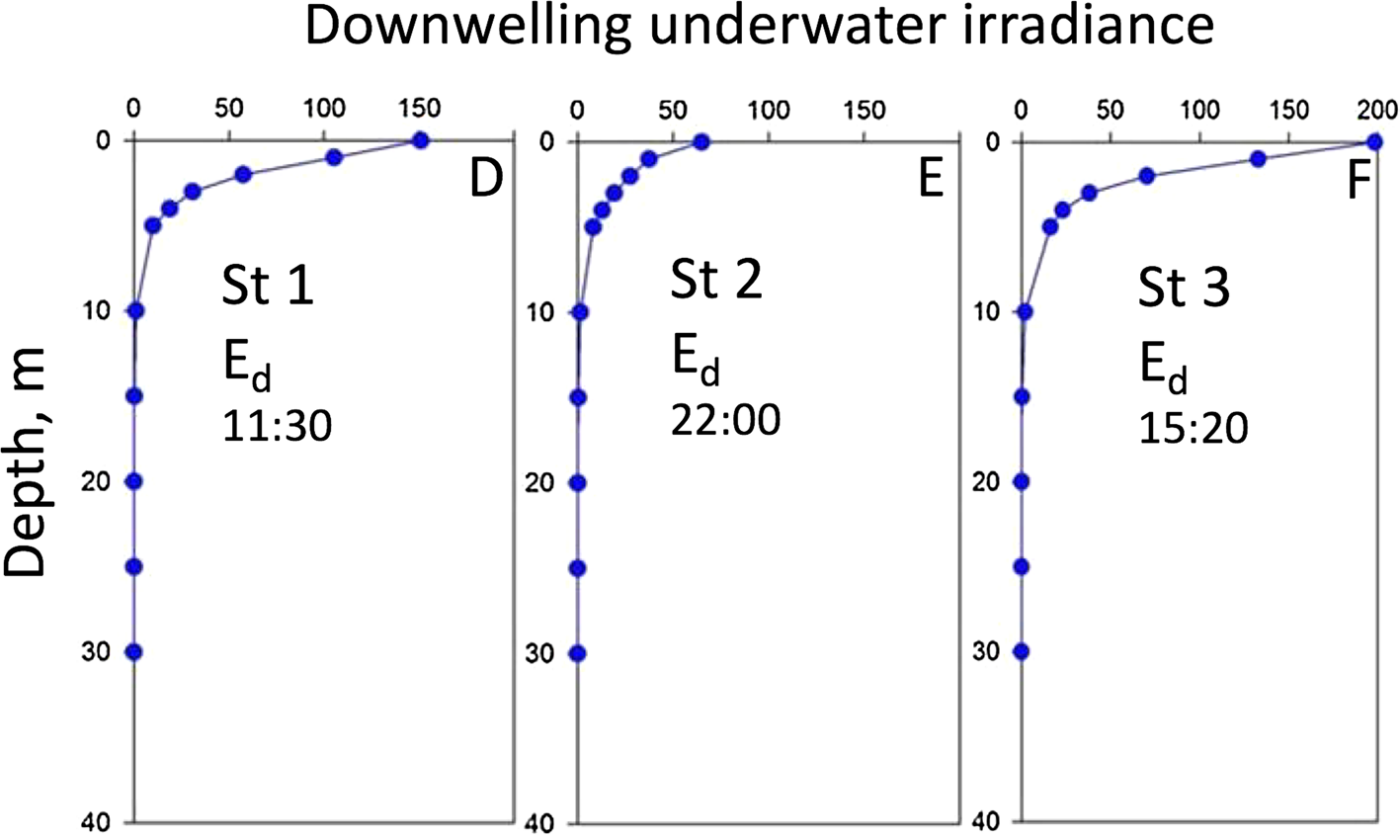
Downwelling irradiance (*E*_d_) as a function of depth at ice station 1–3. The profiles indicate maximum irradiance values (measured from leads)

**Fig. 5:**
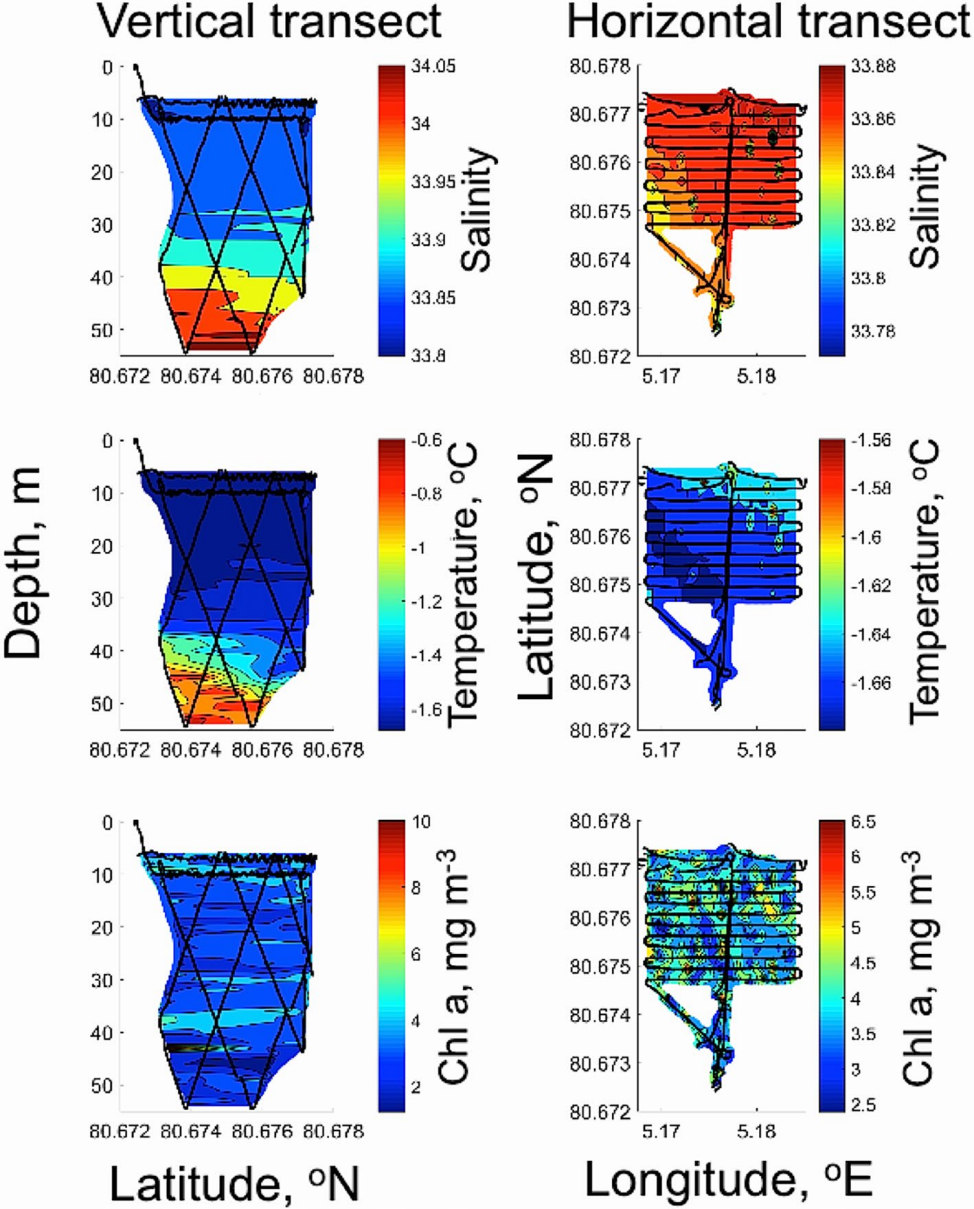
AUV-based vertical (left panels, black lines) and horizontal (right panels, black lines) mapping of phytoplankton biomass ([Chl *a*], lower panel), temperature (mid panel) and salinity (upper panel) of water masses under sea ice at ice station 3. Spatial coverage of Chl *a* (mg m^−3^), salinity (PSU) and temperature (°C) using Remus 100 AUV in vertical zig-zag mode transect covering 16,500 m^2^ and a horizontal grid area at 10 m depth covering 80,000 m^2^ (400 × 200 m)

A REMUS 100 AUV from Hydroid Inc. (Moline et al. 2005) was used under the sea ice at St 3 the 16 May 2010 at 11:00–12:15 local time (Figs. 3, 4, 5, Tables 1, 2, 3). The REMUS 100 was equipped with both ultra short base line (USBL) and long base line (LBL) transducers for positioning and navigation under the ice. With a moving ice field (typically at ≈ 0.5 knots, data from ship log), it was important to ensure that the AUV was navigating relative to the ice features. The average velocity of AUV was 1.5 m s^−1^ (2.9 knots) and the total mission time was 1.15 h with total mission length of 6663 m. Three transponders were placed under the sea ice; two were used for LBL navigation for both the AUV data collection providing vertical profiles and horizontal area coverage at 10 m depth under sea ice. A third transponder (homing transponder) was used for USBL navigation to ensure return of the vehicle to a particular opening in the ice for retrieval.

**Table 2:** Phytoplankton pigment data (HPLC, mg pigment m^−3^) at ice station (st) 1 and 3

Station	Depth (m)	^a^Chl *a* + Phaeo	Chl *a*	Chl*c*_1+2_	Chl b	Fuco	Diadino	Diato	LHP/Chl *a* + Phaeo	LHP/Chl *a*	PPC/Chl *a* + Phaeo	PPC/Chl *a*
1	2	10.8 ± 0.5	9.8 ± 0.5	3.6 ± 0.17		6.0 ± 0.3	1.9 ± 0.1		0.89 ± 0.01	0.98 ± 0.01	0.17 ± 0.01	0.19 ± 0.01
	10	10.7 ± 0.8	9.6 ± 0.6	3.4 ± 0.25		5.6 ± 0.4	1.6 ± 0.1		0.84 ± 0.02	0.94 ± 0.02	0.15 ± 0.01	0.17 ± 0.00
3	10	9.9 ± 0.3	7.9 ± 0.4	2.0 ± 0.43	0.2 ± 0.01	4.0 ± 0.1	1.2 ± 0.07	0.5 ± 0.01	0.63 ± 0.07	0.79 ± 0.1	0.17 ± 0.01	0.22 ± 0.02
	45	7.2 ± 0.6	6.2 ± 0.4	1.9 ± 0.09		3.4 ± 0.1	1.1 ± 0.03		0.75 ± 0.06	0.87 ± 0.06	0.15 ± 0.01	0.17 ± 0.01

^a^Total Chl *a* + Phaeo includes phaeophorbide a, phaeophytin a and chlorophyllide a. Standard error of average (SE, *n* = 3) indicated as ± after [pigment]. Light-harvesting pigments (LHP): Chl *a*, Chl c_1+2_, Fucoxanthin (Fuco). Photoprotective carotenoids (PPC): Sum of diadinoxanthin (Diadino) and diatoxanthin (Diato). Pigment ratios in w:w. Station 3 is the site of AUV survey. Only traces (< 0.02 mg pigment m^−3^) of Chl c_3_ and acyloxy-fucoxanthins were found under ice (indicating low biomass of prymnesiophytes such as *Phaeocystis pouchetii*, data not shown)

**Table 3:** Photosynthetic parameters from ice station 1–3: rETR_max_ ($\phi_{\text{PSII}}^{{\prime }}$ × *E*_PAR_), $\alpha_{\text{ETR}}$, $\phi_{\text{PSII - max}}^{{\prime }}$, *E*_*k*_ (μmol m^−2^ s^−1^) and non-photosynthetic quenching (NPQ) of phytoplankton

Station	Depth (m)	rETR_max_ (± SE)	$\alpha_{\text{ETR}}$ (± SE)	*R* ^2^	$\phi_{\text{PSII - max}}^{{\prime }}$	*E*_*k*_	NPQ *E*_PAR_ = 180	NPQ *E*_PAR_ = 500
1	2	105 ± 7.1	0.69 ± 0.10	0.88	0.60 ± 0.01	152 ± 24.3	0.35 ± 0.03	1.47 ± 0.20
	10	99 ± 7.6	0.64 ± 0.10	0.85	0.62 ± 0.01	155 ± 27.0	0.23 ± 0.07	0.81 ± 0.08
2	2	144 ± 20.8	0.59 ± 0.11	0.79	0.61 ± 0.02	244 ± 57.7	0.23 ± 0.10	1.41 ± 0.30
	10	96 ± 7.7	0.76 ± 0.15	0.80	0.62 ± 0.02	127 ± 27.0	0.89 ± 0.37	1.36 ± 0.30
	40	81 ± 5.4	0.77 ± 0.14	0.84	0.63 ± 0.02	106 ± 20.4	0.58 ± 0.16	2.06 ± 0.47
3	10	89 ± 6.1	0.87 ± 0.17	0.82	0.61 ± 0.01	103 ± 21.2	0.23 ± 0.09	0.82 ± 0.08
	45	68 ± 4.9	0.87 ± 0.20	0.78	0.61 ± 0.01	78 ± 18.9	0.43 ± 0.16	2.02 ± 0.58
Mean		97 ± 8.5	0.75 ± 0.14	0.80	0.61 ± 0.01	138 ± 28.0	0.42 ± 0.18	1.42 ± 0.36

NPQ derived from RLC curves at *E*_PAR_ of 180 μmol m^−2^ s^−1^, close to maximum *E*_*k*_ (mean value of 138 μmol m^−2^ s^−1^) and corresponding light-saturated photosynthesis (*E*_PAR_ of 500 μmol m^−2^ s^−1^). Standard error of average (SE, *n* = 3) indicated as ± after photosynthetic parameters. Station 3 is the site of AUV survey

Progress of the AUV mission was monitored in real time via acoustic modem (hydroid ranger). Two mission plans were run; the first was an under-ice zig-zag pattern generating three vertical transects spanning depths of 5–50 m, the second was an under-ice grid pattern (20 m spacing) at 10 m fixed depth to ensure clearance of pressure ridges. The REMUS 100 was equipped with a Neil Brown CTD for temperature and salinity and depth measurements. In situ [Chl *a*] was measured with a BBFL2 Eco-Puck, with excitation wavelength maximum at 470 nm and corresponding Chl *a* emission measured at 695 nm (Wet Labs, Oregon, USA, calibrated by producer prior to cruise). In addition to these sensors, an upward-looking DC1400 SeaLife digital underwater camera was mounted in the nose of AUV to provide geo-tagged (through time-synchronization with AUV data) qualitative pictures of the underside of the sea ice providing information of light field, ice morphology, water colour (blue, green and white) related to inherent optical properties (IOP) of water and its constituents [phytoplankton, coloured dissolved organic matter and total suspended matter, (Johnsen et al. 2009)]. Vehicle position, time and the lens geometry were used in the mosaic reconstruction of the grid portion of the mission (Fig. 3).

The research vessel “RV *Helmer Hanssen*” (UiT, the Arctic University of Norway) was used for water column profiles of salinity, temperature and depth from 0 to 200 m depth along a transect from stations 1–7 using a Sea-bird 911 CTD (Sea-Bird Electronics, Inc., USA). In addition, the Sea-bird 911 was equipped with a calibrated (prior to cruise) in situ Seapoint (Seapoint Inc, USA) Chl *a* fluorometer (Chl *a*, mg m^−3^), and Niskin water sampler rosette (5 L) for collecting water samples for in vivo analyses of phytoplankton taxa, photosynthetic parameters (onboard research vessel) and for harvesting cells on filters for HPLC pigment characterization (light-harvesting pigments, photoprotective carotenoids and degraded pigments). At each station, immediately after the CTD profiles, vertical net hauls of phytoplankton samples (from below Chl *a* maximum to the surface) were carried out using a phytoplankton net with 20 μm mesh size. Water sample depths are indicated in Tables 1, 2, 3. Phytoplankton samples were kept alive in polyethylene containers at 4 °C. Cell counts and identification were performed on live cells 1–4 h after sampling. No fixatives were added to ensure that fragile naked cells were included in the analysis. Detailed investigation of algal eco-physiology was undertaken at sea-ice sites (St 1–3); note St 3 was also the AUV survey location. This was followed by a transect line southward (St 4–7) with ship-based in situ CTD and Chl *a* fluorescence profiles from surface to 200 m depth (Fig. 1, 2, 3, Table 1).

Downwelling irradiance (*E*_d-PAR_, μmol photons m^−2^ s^−1^), was measured using two different light sensors and data loggers. Irradiance depth profiles (0–40 m at station 1–3) were measured using a calibrated Li-Cor UW1800 2π irradiance (cosine corrected) light collector connected to a Li-Cor 1100 data logger with an underwater cable to 50 m depth (1-m depth increment from surface to 10 m, 5-m depth increments for depths > 10 m, Fig. 4). E_d-PAR_ in open deck incubators (measuring photosynthesis versus irradiance in kelp, data not shown) from a DIVING-PAM irradiance sensor (planar), measuring diurnal time series (15 min between measurements) at 0.5 m depth.

### Identification of phytoplankton groups

Light microscopic (LM) identification of living cells from phytoplankton nets was performed to identify major taxa (class to species level). Cell identification by microscopy (200 and 400 × magnification, using at least 4 subsamples) was further used to separate species into “northern cold water species” and “cosmopolitan species” (Sakshaug et al. 2009b). Ice algae were sampled from the lower part of ice cores and compared with species composition from the water column in order to be able to determine possible origin of the species in the water column according to (von Quillfeldt et al. 2009). In addition, HPLC-derived pigment chemotaxonomy gives information of major pigment groups and pigment-based biomass (including small and fragile cells that are hard to detect using LM), such as sub-groups of Chromophytes (Chl *c*-containing algae), Chlorophytes (Chl *b*-containing classes) and phycobiliprotein-containing cyanobacteria and eukaryotic algae present (Johnsen et al. 1994; Johnsen and Sakshaug 2007; Johnsen et al. 2011a, b; Pettersen et al. 2011; Roy et al. 2011). No traces of prasinoxanthin (marker for small prasinophytes, pigment group 6 in Johnsen and Sakshaug (2007)), phycobiliprotein-containing cryptophytes (alloxanthin) and cyanobacteria (zeaxanthin and b-carotene), affecting photosynthetic parameters ($\phi_{\text{PSII}}^{{\prime }}$) were found (Table 2, see photosynthetic parameters below).

### [Chl *a*] in living cells (in vivo and in situ) and in extracts (in vitro)

The in vitro [Chl *a*], μg L^−1^, measurements were taken from Niskin water samples using both turner designs fluorometer (10-AU-005-CE, Sunnyvale, CA, USA), a Shimadzu Model 1240 UV mini spectrophotometer (see details below) and a high performance liquid chromatograph equipped with a diode array detector (Hewlett Packard series 1100 HPLC) according to Rodriguez et al. (2006). Comparison between in situ fluorescence (CTD and AUV Chl *a* fluorometer) versus analyses on extracts in the lab (fluorometer, spectrophotometer and HPLC) are detailed in discussion. Lab fluorometer (turner designs) was calibrated by the laboratory spectrophotometer using Chl *a* standard from Sigma-Aldrich (C6144 Chl *a*) according to Knap et al. (1996). Due to photoacclimation affected by PQ (photosynthetic quenching, $\phi_{\text{PSII}}^{{\prime }}$) and NPQ (mainly due to photoprotective pigments and low internal pH inside chloroplast at high photosynthetic rates), there is not a direct relationship between in vivo (often called for semi-quantitative [Chl *a*] measurements, (Babin 2008)) and in vitro [Chl *a*]. Therefore, a precise calibration of in situ fluorometers is hard to obtain simply because of light-induced variation of Chl *a* fluorescence emitted from the cells (reviews by Babin 2008; Suggett et al. 2011b; Johnsen et al. 2011a).

Spectrophotometric and fluorometric [Chl *a*] analyses were performed onboard the vessel, whilst HPLC was done at Norwegian University of Science and Technology (NTNU). Water samples for cell identification, pigment (HPLC) and Chl *a* samples from CTD Niskin bottles were taken immediately after the CTD profiles and the AUV survey and were filtered through Whatman GF/F glass fiber filters (25 mm diameter) until the filters were coloured (typically 500 ml water volume due to bloom conditions). Prior to being measured, the algal pigments on filters were extracted in 5 mL of methanol (24 h, 4 °C) and the extracts were refiltered through a 0.2 μm sterile Microsart syringe filter to remove any potential light-scattering particles and debris.

For spectrophotometric [Chl *a*] measurements, filter extracts were measured and the optical density (OD) spectra (300–800 nm, 1 cm cuvette) were obtained, using methanol (MeOH) as blank (OD at 750 nm). The concentration of Chl *a* (mg m^−3^) was calculated using the OD red peak of Chl *a*, at 665 nm in vitro and the extinction coefficient for Chl *a* in methanol at 665 nm (Mackinney 1941), detailed in Chauton et al. (2004).

### HPLC pigment identification and analysis

Glass fiber filters for HPLC analysis of chlorophylls and carotenoids were immediately put in a freezer at − 20 °C during the cruise (1 week) and moved to − 80 °C for 1 month before analysis in laboratory. The frozen filters were used to provide pigment extracts for HPLC (extraction time 24 h at 4 °C in N_2_(g) atmosphere) in 1.6 mL 100% methanol (in 2 mL glass vials). Extracts were refiltered (3 mL syringe with 0.2 μm PTFE filter) prior to being measured. HPLC was used to separate pigments, using a Waters Symmetry C8 column (4.6 × 150 mm, 3.5 μm pore size), a Hewlett Packard Series 1100 HPLC system with a quaternary pump and auto sampler using the procedure outlined in Rodríguez et al. (2006). 77 μL sample was mixed with 23 μL distilled water to increase polarity and better separation of pigments. The mobile phases were methanol:acetonitrile:aqueous pyridine (ratio 50:25:25), acetonitrile:acetone (ratio 80:20) and methanol (for cleaning). The identification of pigments was based on retention time and the OD spectra of the pigment was obtained with a diode array optical density detector (350–800 nm) using our own pigment standards according to Rodríguez et al. (2006).

### Photosynthetic parameters

The photosynthetic parameters from which *E*_*k*_ were calculated, were obtained from RLC (rapid light curve) versus *E* measured using the PAM method using a Phyto-PAM (Walz, Germany) according to Hancke et al. (2008a, b) and Nymark et al. (2009). This method measures the operational quantum yield of charge separations in PSII (Eq. 1) at increasing irradiances (Genty et al. 1989). Three mL seawater was filtered through a 0.2 µm sterile filter (Microsart) and used as a blank. Afterwards, the same cuvette was used for replicate samples (n = 3, water from three different Niskin bottles). Fluorescence cuvette temperature was controlled with a Peltier cell (US-T/S, Walz) mimicking in situ temperature ± 0.2 °C during incubation. Incubation irradiances for RLC were 1, 2, 4, 7, 14, 28, 56, 112, 176, 240, 304, 368, 496 and 624 μmol photons m^−2^ s^−1^, with 30 s incubation time for each E, giving $\phi_{\text{PSII}}^{{\prime }}$ according to Nymark et al. (2009). The maximum quantum yield, was obtained after dark acclimation of cells in 5 min prior to RLC.

The operational quantum yield of charge separations in PSII in actinic light conditions, $\phi_{\text{PSII}}^{{\prime }}$, was calculated according to Genty et al. (1989) (Eq 1):

1 $$\phi_{\text{PSII}}^{{\prime }} = \left( {F_{\text{m}}^{{\prime }} - F_{\text{0}}^{{\prime }} } \right)/F_{\text{m}}^{{\prime }}$$

using the $F_{0}^{{\prime }}$ (“minimum fluorescence”) and $F_{\text{m}}^{{\prime }}$ (“maximum fluorescence” during saturating flash) at different actinic irradiances. Note that cell samples were dominated by chromophytes and that no phycobiliprotein-containing cryptophytes or cyanobacteria, with major Chl *a* emission from PSI, thus affecting $\phi_{\text{PSII}}^{{\prime }}$, were observed in LM or by HPLC pigment chemotaxonomy (Johnsen and Sakshaug 2007).

The irradiance (*E*) and $\phi_{\text{PSII}}^{{\prime }}$ gives the relative electron transfer rate, rETR (Eq. 2):

2 $${\text{rETR }} = E \cdot \phi_{\text{PSII}}^{{\prime }}$$

where the photosynthetic rates (rETR) at a given E was fitted using a non-linear least-squares Marquardt algorithm and the equation from Webb et al. (1974) to provide photosynthetic parameters based on a rETR versus *E* curve (RLC vs. *E* curve, Eq. 3):

3 $${\text{rETR}} = {\text{rETR}}_{\hbox{max} } \left[ {1 - \exp \left( {\frac{ - \alpha \cdot E}{{{\text{rETR}}_{\hbox{max} } }}} \right)} \right],$$

where the photosynthetic parameters are maximum rETR (rETR_max_) and *α*_ETR_ (maximum light utilization coefficient, also called “photosynthetic efficiency”) using Sigma Plot version 11 (Systat Software, USA).

The photosynthetic saturation index, *E*_*K*_ was calculated as (Eq. 4):

4 $$E_{k} = r{\text{ETR}}_{ \hbox{max} } /\alpha_{\text{ETR}},$$

where *E*_*k*_ is in μmol photons m^−2.^s^−1^.

NPQ was calculated using Eq. 5 [Stern–Volmer equation, (Lakowicz 1983)]:

5 $${\text{NPQ}} = \frac{{F_{\text{m}} - F_{\text{m}}^{{\prime }} }}{{F_{\text{m}}^{{\prime }} }},$$

where F_m_ denotes the maximum quantum yield of cells acclimated to dark in 5 min prior to RLC incubations (Maxwell and Johnson 2000; Valle et al. 2014).

## Results

### Remote sensing of phytoplankton spring bloom

The distribution of the phytoplankton spring bloom from satellite images in May 2010 showed elevated [Chl *a*] in the areas south of the Marginal Ice Zone (MIZ, Fig. 1) from 77°N to 80.5°N. The monthly average of satellite-derived [Chl *a*] in the bloom area of 32000 km^2^ varied between 4 and 13 mg Chl *a* m^−3^ (Fig. 1). The ice edge position drawn in Fig. 1 is indicative of its location on May 17th, the day of the southward transect (St 4–7), showing that station 7 was in open waters (sea-ice images May 2010 can be retrieved by www.met.no—daily sea-ice edge analysis). Satellite data (EUMETSAT Ocean and Satellite Application Facility on Ocean and Sea Ice, OSI SAF) on sea-ice edge position at 16 and 17th May also indicated a north to south movement of sea ice from 16 to 17th May in the transect area (see ice edge 16 and 17 May 2010, Fig. 1).

### In situ measurements of salinity, temperature, irradiance and [Chl a] from the research vessel

Salinity, temperature and in situ fluorescence of [Chl *a*] from ice stations (St. 1–6, with St 6 close to MIZ) to open water (St 7) indicate two distinct layers of water in the study area separated vertically by a thermocline and halocline (Fig. 2). The halocline was found at around 40 m depth at St 4–7 and slightly deeper, around 60 m depth, at St 1–3. At low temperatures, water density will follow salinity and therefore a strong pycnocline is also present between the two layers. Below the halocline temperature and salinity values indicate a strong influence of Atlantic water masses with salinity values > 34.7 and temperatures > 0°C. The waters of Arctic origin above the halocline had temperature and salinity characteristic of polar surface water [*T* < 0°C and S 33.8–34.0, (Lind and Ingvaldsen 2012)]. These salinity values (34.0 < S < 34.7) are fresher than that defined for Arctic Water. Maximum concentrations of in situ [Chl *a*] from CTD ranged between 5 and 8 mg Chl *a* m^−3^, and are confined above the halocline going from open to ice-covered waters (Fig. 2). Typically, the Chl *a* maxima were closest to the surface under the sea ice (around 10 m depth) and deeper within the MIZ and open waters (Chl *a* max at 20 m depth). See in vitro [Chl *a*] estimation in the section below.

In situ downwelling irradiance profiles were measured in open leads at ice stations 1–3 (Fig. 3, 4). *E*_PAR_ values at 10 m depth (depth of the AUV horizontal survey, see Fig. 5) at all three stations varied between 1 and 2 μmol photons m^−2^ s^−1^. Importantly, as visualized by the AUV photomosaics (Fig. 3b–c), the leads represent areas of maximum irradiance. Underwater downwelling irradiance at St 1–3 in the upper [Chl *a*] maximum layer at 10 m depth was close to two orders of magnitude lower than the *E*_*k*_ from in vivo photosynthesis from the phytoplankton cells, see photosynthetic parameters below (Tables 1, 2, 3). For logistical reasons, absolute values of *E*_PAR_ from directly underneath sea ice were not possible to obtain. Diurnal surface irradiance south of MIZ 18–19 May at 0.5 m depth (from deck incubators) ranged from 50 μmol photons m^−2^ s^−1^ at midnight to 670 μmol photons m^−2^ s^−1^ at noon.

### Measurements from AUV under sea ice

The undulating AUV transects revealed that under-ice water temperature was generally at − 1.66 °C from 5 to 25 m depth, with corresponding salinity of 33.9 (Fig. 5) characteristic of polar surface waters, PSW (Lind and Ingvaldsen 2012). The vertical distribution of [Chl *a*] (Fig. 5) indicated two maximum layers, one at 0–15 meters depth (with highest concentrations close to the underside of the ice) and one at 30–40 m depth. Values in these layers ranged between 3 and 6 mg Chl *a* m^−3^ (Fig. 5). The horizontal AUV survey (at 10 m depth below the sea ice) indicated patchy, but generally high in situ [Chl *a*] with values ranging from 3.7 to 5 mg Chl *a* m^−3^ (Fig. 3, 5). Both temperature and salinity showed little variability at the 10 m layer (Fig. 5). The horizontal variability in salinity and temperature at 10 m (across 80,000 m^2^) show that the lowest salinities (range 33.85–33.90) corresponded with the lowest temperatures (range − 1.66 to − 1.61 °C). The under-ice photomosaic images from AUV (horizontal mapping grid at 10 m depth at 400 × 200 m area) indicated a patchy and dynamic underwater light field (irradiance values in section above) that varied as a function of ice/snow thickness and leads seen as differences in light intensity and colouration (Fig. 3). The brightest areas (white hues in Fig. 3b–c) were found underneath the ice leads and corresponded to a downwelling *E* of 1–2 μmol photons m^−2^ s^−1^ at 10 m depth and around 20 μmol photons m^−2^ s^−1^ at 5 m. Different hues in blue indicated sea-ice melting ponds (observed at surface), dark green (thick ice) to brighter green to brownish indicating different [Chl *a*] in the water masses and as biofilm of ice algae at the underside of sea ice.

### Phytoplankton biodiversity

**Fig. 6:**
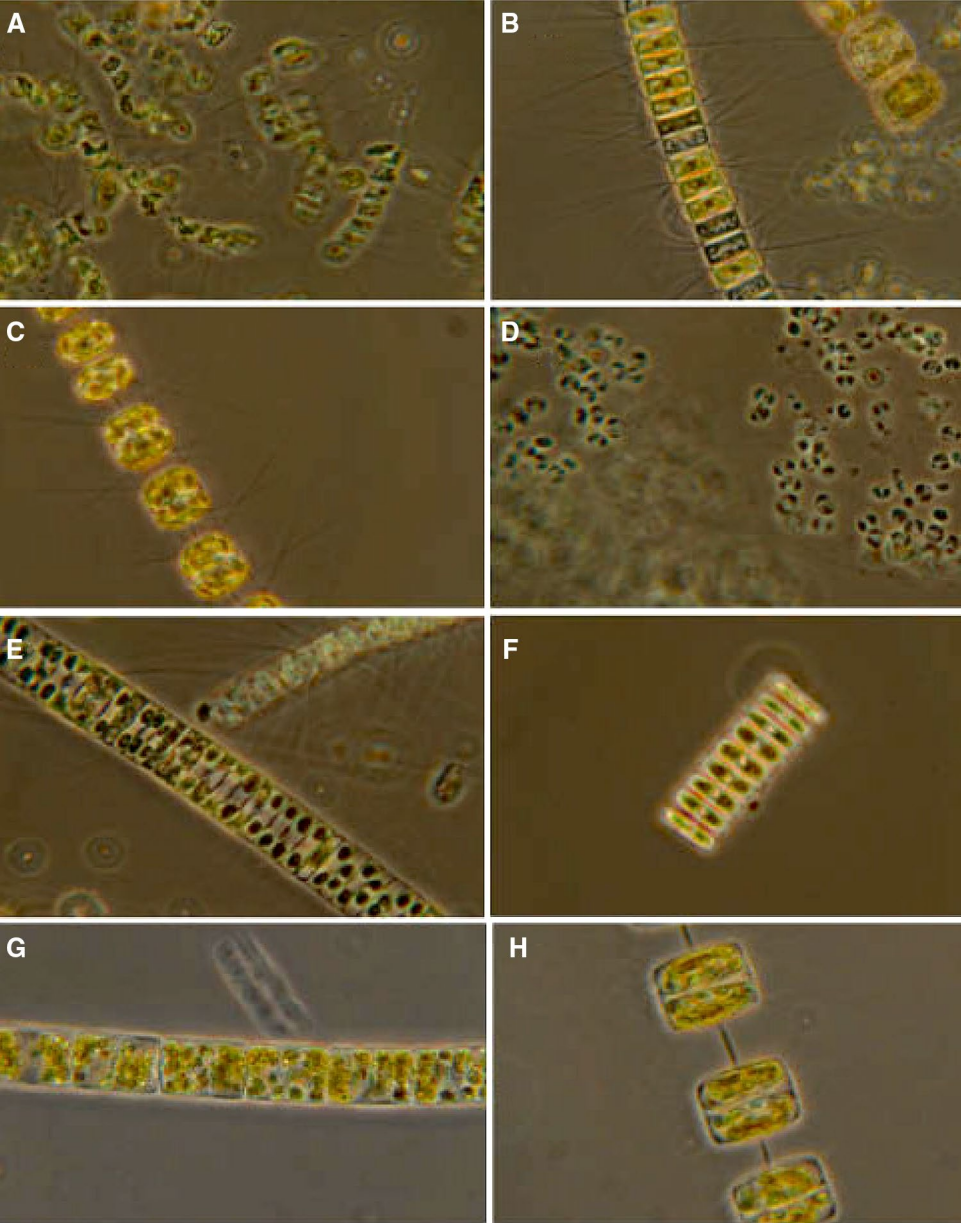
Major phytoplankton species from Ice station 1: *Chaetoceros gelidus (C. socialis,* **a**), *Chaetoceros karinus* (**b**), *Thalassiosira nordenskioeldii* (**c**), *Phaeocystis pouchetii* (**d**), *Fragilariopsis oceanica* (**e**), *Navicula vanhoeffeni* (**f**), *Bacterosira bathyomphola* (**g**) and *Thalassiosira antarctica* var. *borealis* (**f**). Six species of large diatoms made up the largest parts of the biomass. The high cell numbers of *Phaeocystis pouchetii* did not contribute much when looking at [Chl *a*] biomass using its tracer pigments Chl *c*_3_ and 19′-acyloxy-fucoxanthin (Sakshaug et al. 2009b; Johnsen et al. 2011a). Details in Table 4, see also Meshram et al. 2017 using metabarcoding of the microalgae studied in this report

The phytoplankton species composition in the water masses underneath the sea ice and sea-ice bottom samples was different (Table 4, Fig. 6). The phytoplankton cells from the water were dominated by large and typical spring bloom species of diatoms (northern cold or cosmopolitan species) followed by prymnesio-, dino-, prasino- and chrysophytes, while the underside of the ice (from ice core samples) was characterized by typical ice algal species (Table 4). At St 1–3, Niskin water samples and vertical phytoplankton net haul from the under-ice bloom (from 35 m depth to surface) were dominated by a mixture of chain-forming centric (C) and pennate (P) diatoms (Bacillariophyceae), 37 species were identified. Four diatom species comprised the major biomass under ice (St 1–3) and in open waters at St 7 (the 18–20 May), based on relative cell abundance and chemotaxonomic pigment tracers. Abundant diatoms were the centric and spring-forming species *Thalassiosira antarctica* var. *borealis* (Northern cold water to temperate distribution), *T. nordenskioeldii* (Northern cold water to temperate species) and *Chaetoceros socialis* species complex *(*aka *C. socialis*, Cosmopolitan species). Also, the prymnesiophyte (coccolithophore) *Phaeocystis pouchetii* was abundant (many small cells identified), but comprised low biomass indicated by HPLC. The overall biomass was dominated by diatoms, confirmed by high diatom-specific Chl *c*_1+2_ and fucoxanthin concentrations. The chemotaxonomic tracers for *Phaeocystis pouchetii* indicated low biomass relative to the larger diatoms (only traces of Chl *c*_3_ and acyloxy-fucoxanthin found in under-ice samples, data not shown).

**Table 4:** Phytoplankton and ice algae at ice stations (St 1–2)  and open waters (St 7)

Species	Water column St 1	Ice core St 1	Water column St 7	Ice core St 2	Comments*
*Bacillariophyceae*					
*Amphora* sp.			×		Often in ice, but also benthic, P
*Attheya septentrionalis*		×	×	+	Epiphyte on phytoplankton or ice algae, C
*Bacterosira bathyomphala*	+		++		Northern cold water, spring, C
*Chaetoceros borealis*			×		Cosmopolitan, C
*C.* cf. *debilis*	×				Cosmopolitan; C
*C. convolutus*			×		Cosmopolitan, usually more common during summer, C
*C. decipiens*	×		×		Cosmopolitan, season independent, C
*C. furcellatus*	×				Northern cold water, spring, C
*C. socialis* species complex	++		+		Northern cold water, spring, C
*C. karianus*	+		+		Northern cold water, C
*C. teres*	×				Northern cold water to temperate, C
*Cylindrotheca closterium*	×		×	×	Probably cosmopolitan, season independent, P
*Entomoneis* sp.	+	×			Usually spring, often also in sub-ice communities, P
*Eucampia groenlandica*	×		+		Typical northern cold water distribution, C
*Fragilariopsis cylindrus*			×	×	Bipolar? Spring (colonies may also occur in sub-ice communities. Solitary cells have been observed in interstitial communities), P
*F. oceanica*	+		+		Northern cold water, spring (colonies may also occur in sub-ice communities), P
*Hantzschia weiprechtii*		×			Arctic, ice, P
*Navicula directa*			×		Cosmopolitan, P
*N. pelagica*			×		Northern cold water, spring. Usually ice-covered areas, P
*N. septentrionalis*	×		×		Northern cold water, spring, P
*N. vanhoeffenii*	×		+		Northern cold water, spring, P
*N.* sp. 1			×	+++	
*N.* sp. 2				++	
*N.* spp.	+	+	+		Well-developed ice algal communities often consist of many different solitary *Navicula* species, P
*Nitzschia frigida*	×	+	×	++	Arctic, ice, P
*N. laevissima*			×	×	Arctic, ice, P
*Odontella aurita*	×				Cosmopolitan. C
*Pauliella taeniata*			+	×	Northern cold water, spring, P
*Pleurosigma* spp.				×	Both phytoplankton and ice, P
*Porosira glacialis*	+		+		Northern cold water to temperate, spring, P
*Proboscia alata*	×				More common during summer, C
*Pseudogomphonema arcticum*		×	x	×	Epiphyte on phytoplankton or ice algae, P
*Pseudo*-*nitzschia delicatissima*			×x		Cosmopolitan, more common during summer, P
*P. granii*	×		×		Northern cold water to temperate, often in *Phaeocystis* colonies, P
*P. pseudodelicatissima*			×		Cosmopolitan. More common during summer, P
*P. seriata*	×		×		Northern cold water to temperate. More common during summer, P
*Stenoneis inconspicua* var. *borealis*				×	Arctic, ice, P
*Synedropsis hyperborea*		×	×	+	Epiphyte on phytoplankton or ice algae, P
*Thalassiosira antarctica* var. *borealis*	++		++		Northern cold water to temperate, spring, C
*T. bioculata*	×		+		Northern cold water, spring. Often close to ice or part of ice algal communities, C
*T. hyalina*	×		+++		Northern cold water to temperate, spring, C
*T. nordenskioeldii*	++		+		Northern cold water to temperate, spring, C
*Coccolithophyceae*					
*Phaeocystis pouchetii*	++		×		Spring
*Dinophyceae*				×	
*Gymnodinium* spp.	×		×		
*Katodinium* sp.				×	
*Polarella glacialis*			×	+++	Mix of vegetative cells and cysts
*Protoperidinium* spp.	×				More common during summer, but several season independent species
*Prasinophyceae*					
*Pyramimonas* sp.	×			×	
*Chrysophyceae*					
*Dictyocha speculum*			+		Cosmopolitan, season independent
*Dinobryon balticum*			+		More common during summer (sometimes dominating)

Phytoplankton net samples (mesh size 20 μm, haul from 20 m depth to the surface). Ice algal samples from lower 3 cm of ice core. Symbols: × = present, + = regularly occurring, ++ = abundant, +++ = dominant. *Comments include biography, C (centric) and P (pennate) diatoms and own observations. Determination of phytoplankton biogeography follows the reviews (with references therein) in the region by von Quillfeldt (2000), von Quillfeldt et al. 2009 and Sakshaug et al. (2009b)

Ice core samples of bottom side (lower 3 cm, i.e. interstitial community) from St 1 was characterized by ice algae dominated by the pennate sea-ice diatoms *Navicula* spp. and *Nitzschia frigida*. Present in low cell numbers were the pennate sea-ice diatoms *Entomoneis* sp., *Hantzschia weiprechtii, Pseudogomphonema arcticum*, *Synedropsis hyperborea* and the centric diatom *Attheya septentrionalis.* The ice algal community at St 2 was by numbers totally dominated by the dinoflagellate *Polarella glacialis* with many resting cysts, followed by diatoms *Navicula* sp. 1–2 and *Nitzschia frigida* (Table 4).

### HPLC pigment characterization

The HPLC pigment chemotaxonomic signatures from St.1 and 3 revealed dominance of diatoms (Chl *a*, Chl *c*_1+2_, fucoxanthin and diadinoxanthin), verified by microscopy, at all depths. In addition, Chl b indicated the presence of the observed *Pyramimonas* sp., which do not obtain the marker carotenoid prasinoxanthin found in pigment group prasinophyceae II (Johnsen and Sakshaug 2007) (verified by light microscopy), was found at 10 m depth at St 3 (Table 1). Chl *c*_3_ and 19′-acyloxufucoxanthins were found in trace amounts (pigment signature of Chl c_3_-containing prymnesiophytes like *P. pouchetii*, (Johnsen et al. 2011a) at St 1–3 indicating that the biomass (detected as total pigments) of *Phaeocystis pouchetii* was very low (trace amounts) relative to diatoms. Light-harvesting pigments (LHP, chl c_1+2_ and fucoxantin) to Chl *a* ratio (w:w) was close to 1 (Table 2) while photoprotective carotenoids (PPC, diadino- and diatoxanthin) to Chl *a* ratio (w:w) was approximately 0.2. At 10 m depth at St. 3, cells contained both diadino- and diatoxanthin and the PPC to Chl *a* ratio (w:w) was 0.22.

Chlorophyll a to Chl *a* + Phaeo (Chl *a* and its degradation products phaeophorbide a, phaeophytin a and chlorophyllide a) ratio of 0.9 (w:w, Table 2) indicated healthy cells at all depths at St 1 and 3 m, except at 10 m depth at St 3 with Chl *a* to Chl *a* + Phaeo (W:W) ratio of 0.8, indicating peak of bloom to start of post-bloom phase (Johnsen and Sakshaug 1996).

### Photosynthetic parameters

The photosynthetic parameters at ice stations indicate HL-acclimated cells characterized with *E*_*k*_ values ranging from 78 to 244 μmol photons m^−2^ s^−1^ at all depths (Table 3). *E*_*k*_ for 2 m depth was in the range of 105–144 μmol photons m^−2^ s^−1^. Lowest *E*_*k*_ values was found at 45 m depth at St 3 with 78 μmol photons m^−2^ s^−1^ and 103 μmol photons s^−1^m^−2^ at 10 m depth, correspondingly (Table 3). At St 2, the highest *E*_*k*_ of 244 μmol photons m^−2^ s^−1^ was found at 2 m depth and *E*_*k*_ of 127 μmol photons m^−2^ s^−1^ at 10 m depth followed with 106 μmol photons m^−2^ s^−1^ at 40 m depth, comparable to St 3. Correspondingly, *E*_*k*_ at 10-m depth at St 1–3 was remarkably similar with 99 (St 1), 96 (St 2) and 89 (St 3) μmol photons m^−2^ s^−1^, respectively. The maximum photosynthetic rate (rETRmax) was highest in cells from surface and inversely related to *α*. The mean NPQ at light-saturated photosynthesis (*E*_PAR_ of 500 μmol m^−2^-s^−1^) from cells sampled at St. 1–3 was 1.42 ± 25.9% (± CV of mean value, Table 3, see discussion).

### In situ and in vitro [Chl *a*]

**Fig. 7:**
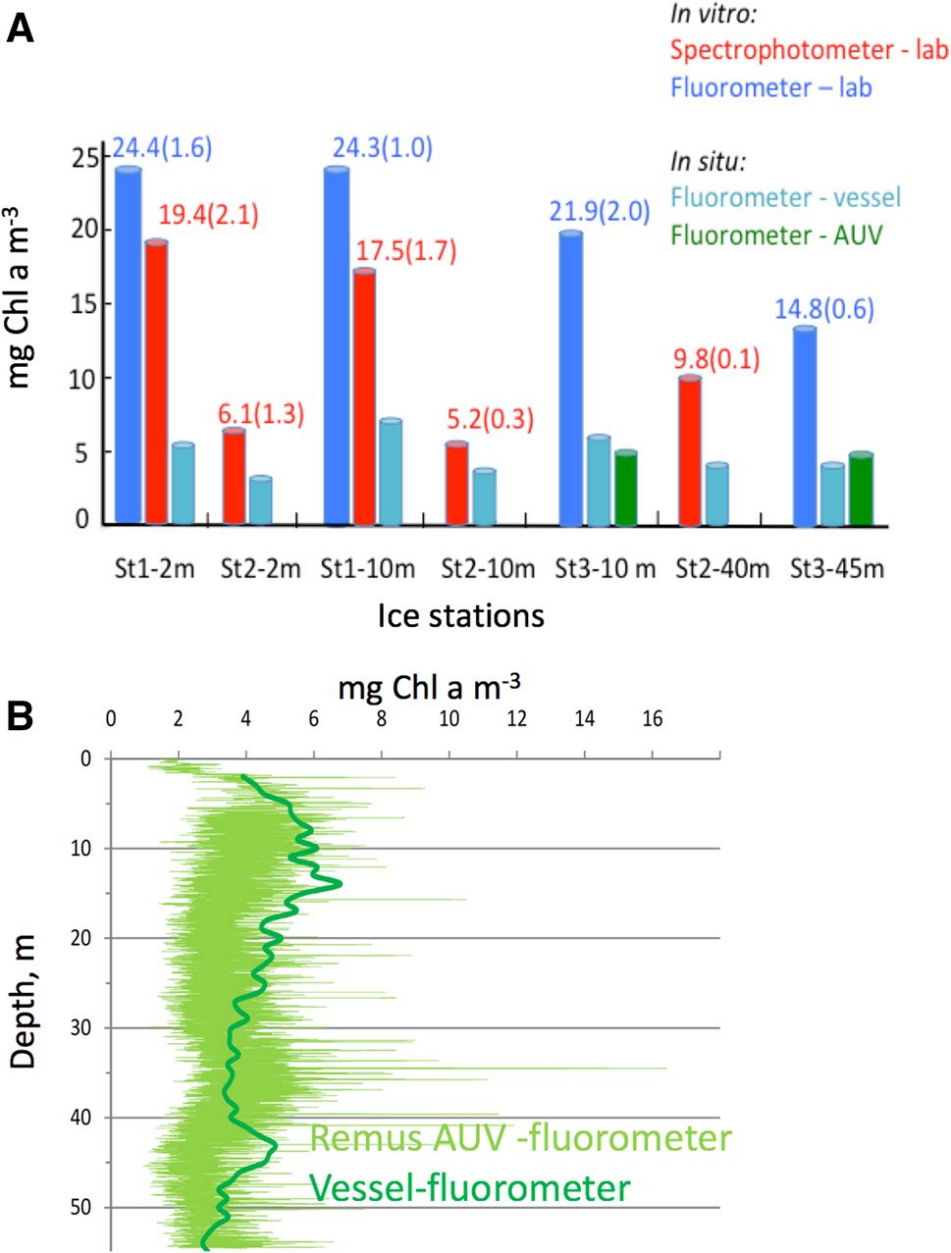
**a** Differences in in situ (living cells) versus in vitro (pigment extracts) [Chl *a*] at ice station (St) 1–3. These differences may mainly be due to quenching of Chl *a* fluorescence due to PQ and NPQ in living cells. Numbers in parentheses indicate ± SE, *n* = 3 for lab-based in vitro measurements. **b** In situ [Chl *a*] from repetitive AUV vertical zig-zag transects at ice station 3 (see also Fig. 5) compared to corresponding ship based vertical [Chl *a*] profile (mean of downcast and upcast in nearby ice-lead)

The mean [Chl *a*] in vitro (pigment extracts of water samples) from St 1–7 relative to in situ [Chl *a*] by AUV fluorometer and CTD fluorometer from vessel, gave an in vitro/in situ [Chl *a*] ratio (w:w) of 2.92 ± 0.86 (*n* = 7) which indicates that in situ [Chl *a*] estimations from healthy and photosynthesizing cells (fluorometer from AUV or ship CTD fluorometer) is only 35% relative to in vitro [Chl *a*], i.e. autofluorescence (pigment extracts) not affected by PQ and NPQ, see discussion (Fig. 7).

## Discussion

In this study we report on the origin and dynamics of an under-ice bloom observed in the Eurasian sector of the Arctic Ocean. Data were obtained simultaneously from three complementary platforms: Remote sensing of ocean colour (satellite), an AUV equipped with environmental sensors and analyses from water samples (from a research vessel). Recent studies in the MIZ north and west of Svalbard (Assmy et al. 2017; Kauko et al. 2017) have reported on blooms developing in situ under a cover of sea ice and snow. We, however, argue that some under-ice blooms may originate from blooms initiated in well-lit open water, and subsequently advected under sea ice. Being able to distinguish between these two scenarios is of vital importance to be able to provide realistic projections of production regimes as sea ice retreats commensurate with a reduction of the Arctic ice cover.

**Fig. 8:**
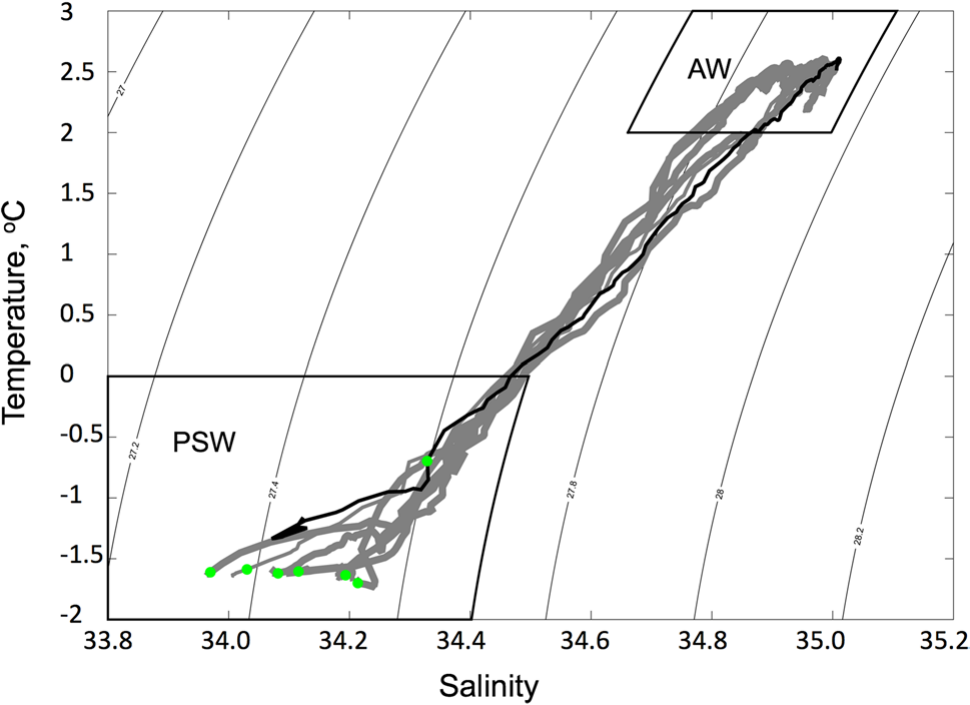
Temperature–salinity (TS) plot of water masses from station 1–7. CTD profile data for each station are shown in grey (MIZ or ice-covered stations) and black (open water station). The [Chl *a*] maximum values are marked by green dots and are found to be consistently in the Polar Surface Water masses (PSW). There are no distinct differences in the TS characteristics of the stations indicating a rather consistent vertical structure of the water—mirrored in Fig. 2. In open water (Station 7), the [Chl *a*] maximum is deeper (25 m) compared to under the ice (~ 6 to 10 m), indicative of vertical redistribution through wind-induced turbulence. The max [Chl *a*] is found at the bottom of the halocline at this station

The oceanographic conditions that were observed during this study are typical of conditions north and south of the MIZ (Fig. 8). Whilst current vectors were not measured directly, the regional flow characteristics (Walczowski et al. 2012) imply a NW transport of the subsurface waters and a corresponding southward movement of sea ice in the region studied in this paper. We are further guided in this assumption based on the observations and model results reported by Assmy et al. (2017) within the region studied here (80.67°–80.18°N and 4.84°–5.31°E) and at a similar time of year (May 2010 vs. May–June 2015). Model data from Assmy et al. (2017) show a strong NW advection of polar surface waters (PSW, mean current velocity ≈ 5 cm s^−1^ at 20–30 m depth) and a NW transport of AW (mean current velocity of 5–10 cm s^−1^) in the location of this study, the SW flank of the Yermak Plateau. Also, sea-ice data during May 2010 in the examined region showed small variations in extent and sea-ice concentration, but it started to disintegrate from 27 May 2010 (Norwegian Meteorological Institute, Svalbard High Resolution Ice Charts; see data base for May 2010 at www.polarview.met.no). Hence, we can conclude that the prevailing oceanographic and ice conditions are supporting our assumption that the observed bloom is of an advective origin (Fig. 8).

In addition to the oceanographic conditions, we examined the taxonomical and eco-physiological phytoplankton status to evaluate whether the bloom was of an advective or an in situ origin. First of all, we note that the phytoplankton species composition was similar from the northernmost ice station to the southernmost station in the open waters south of MIZ (Table 4, see also Meshram et al. 2017 for metabarcoding data of cells close to station 1 in this study). The presence of “Northern cold water” and “temperate” species is typical for spring phytoplankton blooms in northern open waters (Hasle and Syvertsen 1996) and the dominance of centric over pennate spring species indicates that the bloom has been developing for some time (von Quillfeldt 2000; Fragoso et al. 2017). The species composition in the water column was also different from the species composition found on the underside of the ice, which are typical for well-developed ice algal communities (Syvertsen 1991; von Quillfeldt et al. 2009). The species composition of the ice algal community was dominated by diatoms at ice St 1 and dinoflagellates at St 2. This indicates that a “seeding stock” of ice algae was not the origin of the “local bloom”, but, to the contrary, cells in the water masses were brought under the ice by transportation of water masses from the south of MIZ (Fig. 2, 8). In sum, the continuous Chl *a* maxima support the assumption that the bloom observed along the transect was one continuum of PP, rather than two distinct blooms of phytoplankton in open water and under sea ice, respectively. In agreement with the species composition data, the pigment data indicated that the dominating biomass was from diatoms (chl *c*_1+2_ and fucoxanthin) with only traces found of Chl *c*_3_ and acyloxy-fucoxanthin used as chemotaxonomic markers for prymnesiophytes such as *Phaeocystis pouchetii* (Rodríguez et al. 2006; Johnsen and Sakshaug 2007; Pettersen et al. 2011). We also found high *E*_*k*_ values (mean *E*_*k*_ of 138 μmol m^−2^ s^−1^ under sea ice) indicating that the under-ice bloom (ambient *E* < 2 μmol m^−2^ s^−1^ at 10 m depth) may have developed in open water and HL conditions. In addition, the taxonomic composition of the healthy phytoplankton community (seen by $\phi_{\text{PSIImax}}^{{\prime }}$) resembles typical northern planktonic species (different from ice algae found in the sea ice). Finally, a continuum of high [Chl *a*] at sea surface (remote sensing image of Chl *a* for May 2010) from open waters to 31 km north of MIZ, and relatively high amounts of photoprotective carotenoids (PPC: Chl a ratio of 0.17–0.22 at all examined ice stations) also indicates HL-acclimated cells (Rodríguez et al. 2006; Schuback et al. 2016). We discuss each of these lines of evidence in more detail below.

### Photosynthetic parameters

The mean *E*_*k*_ of 138 μmol photons m^−2^ s^−1^ (this study) at ice St 1–3 from 2 to 45 m depth indicate HL-acclimated cells and is comparable with HL-acclimated cells from the Arctic characterized (derived from ^14^C incubations) with *E*_*k*_ > 60 μmol photons m^−2^ s^−1^ (Smith and Sakshaug 1990; Harrison and Cota 1991; Johnsen and Hegseth 1991; Stein and Macdonald 2003; Sakshaug et al. 2009b). These authors provided a range for *E*_*k*_ in LL-acclimated phytoplankton in the Arctic from 0.5 to 60 μmol photons m^−2^ s^−1^ with lowest values for cells acclimated to under-ice irradiances. We obtained mean values of rETR_max,_, *α* and *E*_*k*_ as 97 ± 8.5 (SE), 0.74 ± 0.09 (SE) and 138 ± 28 (SE) μmol m^−2^ s^−1^, respectively (Table 3). Under-ice blooms, dominated by *Phaeocystis pouchetii* in the mid part of the Yermak Plateau with sea ice influenced by leads, leading more light into the under-ice water column, using same PAM method as described here, gave *E*_*K*_ values of 137–584 μmol photons m^−2^ s^−1^ (Assmy et al. 2017). Cultured cells of Arctic diatoms (*Chaetoceros furcellatus* and *Thalassiosira nordenskioeldii*) that were HL acclimated (grown at *E* of 400 μmol photons m^−2^ s^−1^ at 24 and 12 h day length) and LL acclimated (grown at *E* of 25 μmol photons m^−2^ s^−1^ at 24 and 12 h day length) isolated from the same area in the Barents Sea (78°N, 30°E) obtained typically an *E*_*k*_ of 60 (12 h day length) to 70 (24 h day length) and 31 (12 h) to 35 (24 h) μmol photons m^−2^ s^−1^, respectively (Sakshaug et al. 1991, review in Sakshaug et al. 2009b). Comparing ^14^C data with ETR data is difficult and currently not resolved. Photosynthetic ^14^C- incubators were widely used until the end of the 1990’s, typically using the “Photosynthetron” photosynthesis versus irradiance curve (*P* vs. *E* curve) technique, often with 20 min E_PAR_ incubation time (Lewis and Smith 1983), compared to rETR data derived from short incubation time experiments using PAM or FRRF (0.2–5 min incubation time per *E*_PAR_ to provide ETR vs. *E* curves (Suggett et al. 2011a; Schuback et al. 2016)). There are still several challenges in understanding the variables affecting photosynthetic rates such as differences in incubation time, absorbed quanta utilized by PSII, diurnal oscillations of photosynthetic parameters and lastly, how to convert ETR rates (Chl *a* fluorescence from PSII) into C-fixation rates (Johnsen and Sakshaug 2007; Hancke et al. 2008a, b; Schuback et al. 2016). What we do know is that the rapid light curves (RLC) give higher *E*_*k*_ values than *P* versus *E* curves derived from PAM with 5 min incubation time or *P* versus *E* curves from “Photosynthetron technique” making comparison of photoacclimation (comparing *E*_*k*_ values) difficult. PAM- and O_2_-electrode-derived *E*_*k*_ values for diatoms may be similar to short time ^14^C incubations (20–30 min) using the “Photosyntethron” technique (Lewis and Smith 1983; Johnsen and Hegseth 1991) compared to PAM and bio-optical-derived data (Hancke et al. 2008a). The findings of Assmy et al. (2017), in the middle part of Yermak plateau (May–June 2015) with a significantly lower current speed than this study from S-Yermak plateau (May 2010), found E_k_ values ranging from 137 to 584 μmol photons m^−2^ s^−1^ of blooms dominated by *Phaeocystis pouchetii*, under leads in pack ice based on RLC curves (same method as used in this study). However, it should be noted that *Phaeocystis pouchetii* generally obtain higher *E*_*k*_ that is 2–3 times higher than diatoms under same ambient light conditions (data from lab cultures and in situ data from the Barents Sea, (Sakshaug et al. 2009b)).

A study comparing photoacclimation status (*E*_*k*_) in three classes of phytoplankton as a function of temperature (0–35 °C in diatoms, prymnesiophytes and dinoflagellates) using ETR (PAM), O_2_ evolution and ^14^C incubation, gave *E*_*k*_ values in agreement with each other from 0 to 15 °C for diatoms (Hancke et al. 2008a). In the latter study, the O_2_ method gave generally the highest *E*_*k*_ values when looking at all three phytoplankton classes, followed by rETR and ^14^C-method (pigment group dependent). Closest agreement was found between *E*_*k*_ derived from O_2_ and rETR data, with ^14^C generally giving lowest *E*_*k*_ values. Since most *E*_*k*_ values in literature are based on ^14^C method, a direct comparison may be hard to interpret. Still, our *E*_*k*_ data indicate that the diatom cells are HL acclimated and is in the low range of under-ice *E*_*k*_ findings by Assmy et al. (2017) of *Phaeocystis pouchetii*-dominated bloom, using the same RLC method as in this study. For details of underwater irradiance in similar type of sea ice at same time of the year, Kauko et al. (2017) observed similar light conditions as in this study.

The NPQ of in vivo Chl *a* fluorescence is a physiological response related to the lowering of pH (during photosynthesis) in the thylakoids inside the chloroplasts which induces de-epoxidation of diadinoxanthin to diatoxanthin in diatoms and prymnesiophytes (coccolithophytes) through the “xanthophyll cycle” of PPC (Brunet et al. 2011; Valle et al. 2014), state II–I transitions between PSII and PSI (occurs mainly in green algae and phycobiliprotein-containing phytoplankton) and finally photoinhibitory quenching (Govindjee 1995; Falkowski and Raven 1997). Regarding NPQ, we found no pigment groups (green algae and cryptophytes) that may possess state II–I transitions inducing further lowering of fluorescence signal emitted from cells (Brunet et al. 2011; Johnsen et al. 2011a; Schuback et al. 2016) and no photoinhibitory irradiances under ice were observed (*E*_PAR_ at 10 m depth under sea ice ranged from 1 to 2 μmol m^2^ s^−1^) and these two factors cannot explain the high *E*_*k*_ and high PPC indicating HL-acclimated cells (Table 3). This leaves the reduction of Chl *a* fluorescence emitted from cells in situ (Fig. 7) by the two remaining NPQ mechanisms: first, the pH gradient across the thylakoid membrane (causing acidic conditions when photosynthetic rate is high and thus reduction of fluorescence emitted from the cells). Secondly, the corresponding lowering of pH inducing the xanthophyll cycle of diadino- and diatoxanthin (acting as radiators, emitting absorbed light energy as heat) and corresponding quenching of Chl *a* fluorescence, which requires HL conditions far above *E*_*k*_ level, which were not observed under the sea ice (Table 2, Brunet et al. 2011). This is another indication that the cells found under the sea ice may have been advected and/or covered by sea ice from well-lit, ice-free surface waters from the south of MIZ. A lack of saturation in NPQ was observed at ice St 1–3 dominated by diatoms, despite photophysiologial stress on cells during RLC exposed to high-light conditions up to 300 times higher than ambient under-ice irradiance (Table 3). This suggest the presence of HL-acclimated cells still with the capacity of photoprotective downregulation as shown in Lavaud and Kroth (2006) and Schuback et al. (2016).

### Photoprotective carotenoids and light-harvesting pigments

The phytoplankton sampled under the sea ice, characterized by low under-ice *E*_PAR_ (1–2 μmol photons m^−2^ s^−1^ at 10 m depth) was not acclimated to the ambient light intensities. High cellular contents of diadinoxanthin, indicating ML- (medium) to HL-acclimated cells, were found at St 1 and 3 (Table 2). Cells obtained diadinoxanthin to Chl *a* ratios (w:w) ranging from 0.17 to 1.19 typical for ML-HL-acclimated cells (Johnsen and Sakshaug 1993; Rodríguez et al. 2006; Schuback et al. 2016). Note that conversion (epoxidation) from diatoxanthin to diadinoxanthin in dim/dark–light conditions due to handling of water samples and filtering of cells in dim light may happen in seconds and therefore the sum of diadino- and diatoxanthin would often be the best measure of photoacclimation status (Rodríguez et al. 2006; Brunet et al. 2011). Significant amounts of diadinoxanthin, characteristic for HL-acclimated phytoplankton (Johnsen et al. 1994; Rodríguez et al. 2006; Johnsen and Sakshaug 2007; Brunet et al. 2011), can be de-epoxided to diatoxanthin as a response to rapid (seconds to hour) increase of irradiance (Brunet et al. 2011).

### In vitro versus in vivo/in situ Chl *a* fluorescence

Caution regarding in situ (or in vivo) versus in vitro [Chl *a*]-based fluorometry for interpretation of data due to PQ and NPQ are discussed below. The observed bloom reached 20 mg Chl *a* m^−3^ (in vitro), which can be regarded as a high density bloom (Roy et al. 2011). In contrast, the corresponding in situ [Chl *a*] ranged under the sea ice St 1–4 from 4 to 8 mg Chl *a* m^−3^ and with the highest [Chl *a*] centred at St 5 (8 mg Chl *a* m^−3^ maximum at 10 m depth, Fig. 2, 7). In addition, at St 3, the AUV survey indicated patchy distribution of [Chl *a*] under the sea ice (Fig. 5), with a maximum [Chl *a*] found at 10 and 38 m depth (vertical surveys) and at 10 m depth (horizontal survey) ranging between 3 and 6.5 mg Chl *a* m^−3^. Differences between in situ and in vitro estimations of [Chl *a*] may be, in addition to patchiness of phytoplankton biomass, due to quenching of Chl *a* fluorescence in living cells and differences in methodology (HPLC with separation of Chl *a* and its degradation products, spectrophotometer and bench fluorometer). In living cells (in vivo) *ϕ*_F_ denotes the fraction of light absorbed and utilized by PSII (Photosystem II, the oxygen evolving site) that is converted to Chl *a* fluorescence [emission detected at 685 nm in most fluorometers, (Babin 2008; Suggett et al. 2011b)]. The in vivo (including in situ) Chl *a* fluorescence is different from in vitro measurements because living cells change *ϕ*_F_ by two major processes defined as photochemical quenching (PQ) and NPQ. The PQ of Chl *a* fluorescence is a function of the fraction of closed reaction centres of PSII, $\phi_{\text{PSII}}^{{\prime }}$, see M & M (reviewed in Babin 2008; Johnsen et al. 2011a; Suggett et al. 2011a; b). In addition, the in vitro versus in situ [Chl *a*] measurements can be used to evaluate the effect of photosynthetic absorption and utilization of ambient irradiance by looking at PQ and NPQ. The highest *ϕ*_F_ for Chl *a* fluorescence will be found in vitro (extracted pigments) since the Chl *a* has lost its apoproteins and is non-functional in photosynthesis (the rate constant for photochemistry is 0) and a value of *ϕ*_F_ of 30% can be obtained (autofluorescence, Owens 1991; Johnsen et al. 2011a). In living and photosynthetically active cells, *ϕ*_F_ in situ is typically 0.5–5% re-emission per quanta absorbed, about 70% of absorbed quanta will be lost as thermal decay (heat), and the rest, 25–30%, will be used in photochemistry affecting PQ (Falkowski and Raven 1997; Johnsen et al. 2011a; Suggett et al. 2011a). For diatoms a further reduction of Chl *a* fluorescence emitted from living cells will be due to NPQ processes, mainly due to low pH (H + produced by the water-splitting complex in PSII, creating acidic condition and reducing the fluorescence emitted from cells) and light absorption and corresponding heat radiation from PPC thus not sending the absorbed energy further to PSII (reviews in Govindjee 1995; Falkowski and Raven 1997; Babin 2008; Brunet et al. 2011; Johnsen et al. 2011a and Suggett et al. 2011a).

By comparing in vitro to in situ [Chl *a*] ratios (w:w, with in situ and in vivo Chl *a* fluorescence highly sensitive to PQ and NPQ) from research vessel and/or AUV fluorometer we obtained ratios of 1.3–4.4 with a mean ratio of 2.92 ± 0.86 (*n* = 7), indicating a significant loss in *ϕ*_F_ due to PQ and NPQ (Fig. 7). The mean $\phi_{\text{PSII}}^{{\prime }}$ of 0.61 ± 1.3% (all water samples from st 1–3) at lowest RLC incubation (Phyto-PAM) irradiance of 1 μmol photons m^−2^ s^−1^ (similar to the light intensities observed under sea ice) indicated healthy cells with a high fraction of open reaction centres to perform photosynthesis, i.e. cells far from light saturation in the under-ice ambient light (Table 3, Hancke et al. 2008a, b).

In vitro analyses provide the most accurate measurements of [Chl *a*] and is often based on spectrophotometric measurements (high precision and accuracy, but low sensitivity). In contrast, Chl *a* fluorometers have about 100–200 times higher sensitivity than spectrophotometers, but are dependent on calibration from spectrophotometers using Chl *a* standards (Johnsen and Sakshaug 2000; Roy et al. 2011; this study). Note that fluorometer readings of Chl *a* are highly influenced by the different fluorescent fractions of Chl *a* such as its degradation products chlorophyllide *a* and phaeopigments (Johnsen and Sakshaug 2000; Roy et al. 2011). The Chl *a* fluorescence is also in vitro highly pH dependent and adding of acid may be used to look at phaeopigments using broadband fluorometers, spectrofluorometers or spectrophotometers (Holm-Hansen et al. 1965; Johnsen and Sakshaug 2000; Roy et al. 2011). Many put in 1–2 drops of HCl in different volumes of pigment extracts to estimate the fraction of “phaeopigments” and these readings are highly depended on pH giving different degradation products of Chl *a* with different emission strength making quantification difficult (Johnsen and Sakshaug 1993).

## Conclusions

A coordinated campaign with the main aim of studying the dynamics and origin of an under-ice pelagic bloom north of Svalbard was carried out in May 2010. A continuum of high [Chl *a*] above the halocline was found along the transect from open water and 31 km into the pack ice. The bloom was characterized with high-light-acclimated phytoplankton cells (based on E_k_, PPC and NPQ) along the entire transect, including the ice stations deepest into the sea ice. Typically, the Chl *a* maximum was closer to the surface under the ice (around 10 m depth) and deeper within the MIZ and open waters (20 m depth). Also, the, species composition was similar at northernmost ice stations (31 km into the sea ice) and south of the MIZ (open waters). We conclude that the bloom observed beneath the sea ice North of Svalbard in May 2010 was an advective phenomenon, with cells that had developed in open water further South. This advective mechanism is in contrast to the in situ under-ice bloom scenarios elucidated by Arrigo et al. (2012) and Assmy et al. (2017) where under-ice, advective flow was considerably weaker compared to our study. Knowledge about the origin and dynamics of pelagic blooms in the MIZ and PIZ is essential in order to provide realistic projections of production regimes in a future warmer, and hence less ice-covered Arctic. This study also imply that comparisons/calibrations between different P vs E methodologies and incubation times are highly needed for a common currency to compare between laboratory and in situ measurements as outlined by Schuback et al. (2016).
